# Altered Gene Expression and DNA Damage in Peripheral Blood Cells from Friedreich's Ataxia Patients: Cellular Model of Pathology

**DOI:** 10.1371/journal.pgen.1000812

**Published:** 2010-01-15

**Authors:** Astrid C. Haugen, Nicholas A. Di Prospero, Joel S. Parker, Rick D. Fannin, Jeff Chou, Joel N. Meyer, Christopher Halweg, Jennifer B. Collins, Alexandra Durr, Kenneth Fischbeck, Bennett Van Houten

**Affiliations:** 1 Laboratory of Molecular Genetics, National Institute of Environmental Health Sciences, Research Triangle Park, North Carolina, United States of America; 2 Neurogenetics Branch, National Institute of Neurological Disorders and Stroke, Bethesda, Maryland, United States of America; 3 Expression Analysis, Durham, North Carolina, United States of America; 4 Laboratory of Toxicogenomics, National Institute of Environmental Health Sciences, Research Triangle Park, North Carolina, United States of America; 5 Nicholas School of the Environment, Duke University, Durham, North Carolina, United States of America; 6 Centre de Recherche de l'Institut du Cerveau et de la Moelle Epinière, Université Pierre et Marie Curie, Paris, France; 7 Département de Génétique et Embryologie, Hôpital Pitié-Salpêtrière, Paris, France; 8 Department of Pharmacology and Chemical Biology, University of Pittsburgh School of Medicine, Pittsburgh, Pennsylvania, United States of America; 9 University of Pittsburgh Cancer Institute, Hillman Cancer Center, University of Pittsburgh, Pittsburgh, Pennsylvania, United States of America; The Hospital for Sick Children and University of Toronto, Canada

## Abstract

The neurodegenerative disease Friedreich's ataxia (FRDA) is the most common autosomal-recessively inherited ataxia and is caused by a GAA triplet repeat expansion in the first intron of the frataxin gene. In this disease, transcription of frataxin, a mitochondrial protein involved in iron homeostasis, is impaired, resulting in a significant reduction in mRNA and protein levels. Global gene expression analysis was performed in peripheral blood samples from FRDA patients as compared to controls, which suggested altered expression patterns pertaining to genotoxic stress. We then confirmed the presence of genotoxic DNA damage by using a gene-specific quantitative PCR assay and discovered an increase in both mitochondrial and nuclear DNA damage in the blood of these patients (*p*<0.0001, respectively). Additionally, frataxin mRNA levels correlated with age of onset of disease and displayed unique sets of gene alterations involved in immune response, oxidative phosphorylation, and protein synthesis. Many of the key pathways observed by transcription profiling were downregulated, and we believe these data suggest that patients with prolonged frataxin deficiency undergo a systemic survival response to chronic genotoxic stress and consequent DNA damage detectable in blood. In conclusion, our results yield insight into the nature and progression of FRDA, as well as possible therapeutic approaches. Furthermore, the identification of potential biomarkers, including the DNA damage found in peripheral blood, may have predictive value in future clinical trials.

## Author Summary

Friedreich's ataxia is an inherited disease that causes progressive damage to the nervous system and affects the muscles and heart. The disease is caused by a defect in the frataxin gene, which is involved in iron homeostasis and likely protects against reactive oxygen species. In order to identify mechanisms involved in the nature and progression of the disease, we performed transcriptional profiling and measurements of mitochondrial and nuclear DNA damage on blood cells from FRDA patients. Transcriptional profiling was performed on blood samples from a cohort of 28 children compared to a control group. These data were then validated with a cohort of 14 adults with FRDA compared to a second independent control group. DNA damage was assessed on the blood samples from the 28 FRDA children, plus an additional 19 affected children, by quantitative PCR (QPCR). Transcriptional profiling revealed changes in gene expression consistent with the presence of genotoxic stress in FRDA patients. This finding was supported by the direct evidence that FRDA patients accumulated significantly higher levels of mitochondrial and nuclear DNA damage as compared to controls. The identification of potential biomarkers, including the DNA damage found in peripheral blood, may help identify therapeutic approaches for this devastating disease.

## Introduction

Friedreich's ataxia (FRDA; OMIM# 229300) is the most common autosomal-recessively inherited ataxia beginning in childhood and leading to death in early adulthood. Patients exhibit neurodegeneration of the large sensory neurons and spinocerebellar tracts, along with variable systemic manifestations that include hypertrophic cardiomyopathy, scoliosis, and diabetes mellitus (see http://www.ncbi.nlm.nih.gov/entrez/dispomim.cgi?id=229300).

FRDA results from the partial loss of frataxin (*FXN*; Entrez Gene ID 2395), a small nuclear encoded 18-kDa protein targeted to the mitochondrial matrix [1]. A GAA triplet repeat expansion in the first intron impairs transcription of frataxin, resulting in a significant reduction in mRNA and protein levels [2]–[4]. The exact physiological function of frataxin is still unclear, but it has been shown to bind iron and play a role in iron-sulfur cluster (ISC) assembly [5],[6]. A decrease in frataxin may also increase reactive oxygen species (ROS) produced by increases in bioavailable iron [5], [7]–[10] and the lack of iron detoxification [11]. The conclusions of several studies indicate that a defect in ISC assembly is the primary event in frataxin-deficient cells [5], [12]–[14] and that ROS production is a secondary event [8],[15]. Napoli et al. [12] believe the dysfunction of biosynthesis of mitochondrial iron-sulfur clusters, and deficiency of ISC enzyme activity, produces a defect in heme, which in turn causes a loss of cytochrome C. Impairment of electron transport activity results in higher levels of ROS production [14], and according to Napoli et al. [12], it is the decrease in cytochrome C that leads to the unchecked increase in production of mitochondrial ROS in Friedreich's ataxia patients. This hypothesis is further supported by studies of yeast strains with reduced frataxin, which accumulate mitochondrial iron and generate reactive hydroxyl radicals that damage membranes, proteins, and mitochondrial DNA (mtDNA), ultimately resulting in the decreased capacity for ATP synthesis through impaired oxidative phosphorylation [15],[16]. Moreover, evidence consistent with nuclear DNA (nDNA) damage is demonstrated by decreasing the levels of frataxin in a *RAD52* (854976) double-strand break repair deficient yeast strain, which results in rapid G2/M cell cycle arrest [16].

In FRDA patients, iron deposition is observed in neuronal and myocardial cells and suggests the potential for free radical damage [17],[18]; however, we note that the case for oxidative stress has been somewhat controversial. Cell models support sensitivity to oxidative stress, and patient studies have found markers of oxidative stress [7],[19],[20], but a conditional knock-out (KO) mouse model did not show oxidative stress, or improvement, when overexpressing superoxide dismutase (SOD) [21]. Recent studies have also failed to replicate the previous marker data [22],[23]. Therefore, it is important to examine other markers of oxidative stress by more sensitive and specific means, such as testing for mtDNA damage in the patient. There is good evidence to suggest that hypertrophic cardiomyopathy, which leads to the death of most FRDA patients, is probably a consequence of iron-catalyzed Fenton chemistry causing damage to mitochondrial macromolecules followed by muscle fiber necrosis and a chronic reactive myocarditis [24]. More work is needed to understand the causes of the pathobiology associated with the progression of FRDA.

While genome-wide scans in frataxin-deficient model organisms and mammalian cells have previously been published [15], [25]–[27], we report the first study involving transcription profiling of total blood from children with FRDA. These gene expression data were further validated in a second cohort of adults with FRDA, who were compared to an independent group of controls. Importantly, we observed previously unreported signatures of gene expression associated with DNA damage responses. Based on these results, we further analyzed patient mitochondrial and nuclear DNA from peripheral blood and detected high levels of damage as compared to control samples. These results provide insights into the nature of the disease and a working model for frataxin deficiency in humans.

## Results

### Microarray analysis of global gene expression in total blood from children with FRDA

We set out to identify mechanisms involved in the nature and progression of Friedreich's ataxia by analyzing global gene expression changes in blood samples from 28 FRDA children involved in an idebenone clinical trial [22] (Table S1). Blood samples were collected from the children prior to the administration of idebenone. The protocol only allowed one 8.5 ml sample of blood for the RNA isolation, which resulted in a limited amount of RNA for this study. Furthermore, control unaffected children were not included in this clinical trial; therefore, we used the youngest control adults available from an NIEHS sponsored study [28] for the gene expression analysis (Table S1).

**Figure 1:**
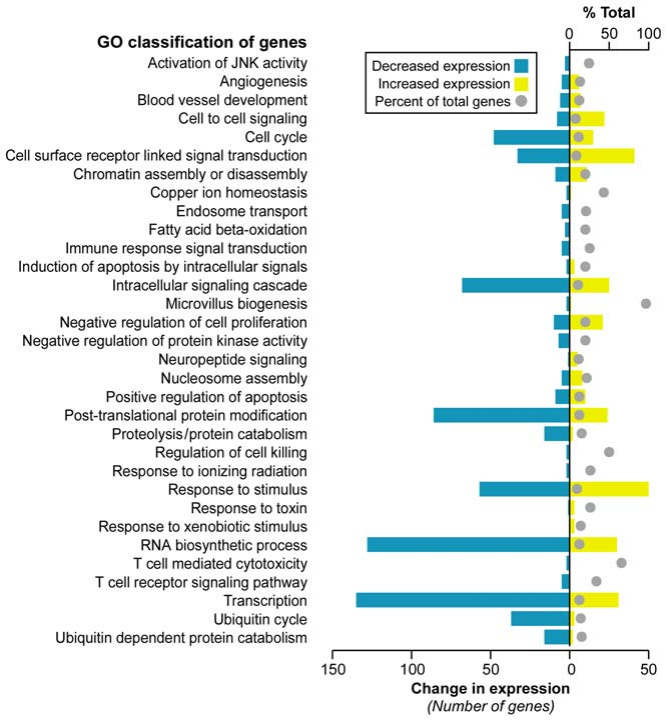
Selected gene classifications according to biological processes. Significantly regulated genes (SAM FDR<0.023%; n = 1,370) from FRDA children versus healthy young adults were grouped according to the gene ontology category of biological process (*p*≤0.05 in *updown*, *over*, or *under* [expression] output lists). The percent of total (displayed with a gray ball) is based on the number of significantly changed genes out of the total number of genes assigned to each gene ontology term. The change in expression for each GO term is depicted in yellow for upregulation and blue for downregulation.

Significance Analysis of Microarray (SAM) [29] identified 1,370 differentially expressed genes at a false discovery rate (FDR) less than 0.023% (Dataset S1). A majority of genes, 899, were downregulated in FRDA compared with control, while 471 genes were upregulated. We further investigated whether these altered transcripts (FDR<0.023%) were associated to specific gene ontology (GO) terms, at *p*≤0.05, in order to assess the global impact of FRDA on gene expression. This analysis identified significant functional groups that included apoptosis signaling, transcription/RNA processing, cell-cell signaling, cell cycle, ubiquitin cycle, proteolysis/protein catabolism, response to stimuli, and fatty acid beta-oxidation (Figure 1).

Although age-matched control children were not available for this study, we decided not to use data uploaded to GEO by other laboratories. It is important to minimize cross-platform differences, which can give rise to large technical variation that may obscure small biological differences in blood cells. Therefore, the controls we used were gene expression profiles from young adults processed in our same facility, on the same oligonucleotide chip design, by the same operator. However, we did assess what effect age might have on gene expression by using SAM to test for any association. This analysis was performed in the controls, and age was dichotomized by comparison to the median age of the controls. Age was not found to be associated with any gene expression value after multiple testing correction (min *q* = 0.47); thus no age-specific gene expression changes were discerned in our control group.

### Gene Set Analysis reveals common gene signatures to genotoxic stress responses in children and a validation cohort of adults with FRDA

**Figure 2:**
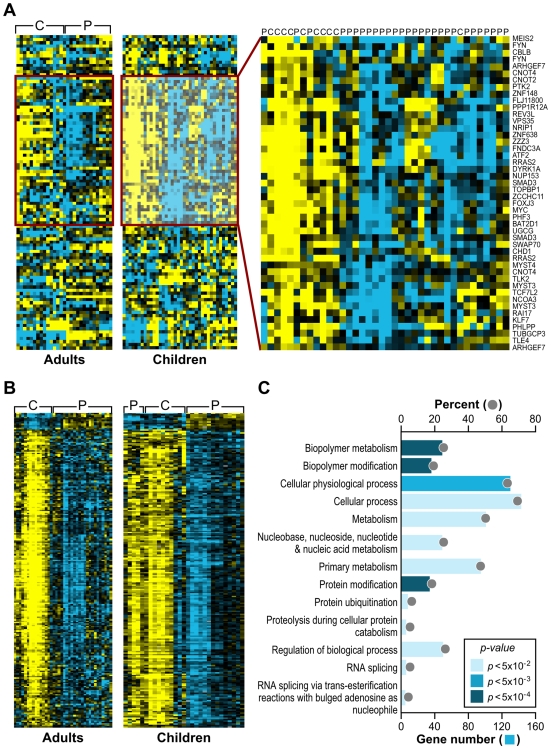
Analogous gene expression responses in blood from children and adults with FRDA. (A) A common gene signature is representative of a genotoxic stress response found by Gene Set Analysis. Twenty-three genotoxic stress response gene sets were searched for common genes. This heat map, generated by unsupervised clustering, displays the genes present in at least 6 of the 23 gene sets and compares transcript levels between FRDA patients, the adult and children cohorts, and controls. Yellow = upregulated; Blue = downregulated. C = Control; P = Patient. Note that while three patients and one control did not segregate with their respective groups, all adult patients and controls clustered in two separate groups in this unsupervised clustering. (B) Heat-map generated by unsupervised clustering of FRDA and control samples, which displays the overlap of significantly differentially expressed genes (SAM FDR<0.023%; n = 228) in the FRDA children and FRDA adults (overlap *p*≤0.007). C = control; P = patient. (C) A selected list of significant GO groups representing the overlap gene list described in (B). All controls used for comparison to the FRDA children are young adults (see Table S1). The percent of total (displayed with a gray ball) is based on the number of significantly changed genes out of the total number of genes assigned to each gene ontology term. The gene number for each GO group is shown with a blue bar, the intensity of which is indicative of the *p*-value.

As an alternative approach to gene ontology enrichment analysis, the microarray data for children with FRDA were further analyzed by employing Gene Set Analysis (GSA), a tool that uses predefined gene sets to identify significant biological changes in microarray datasets [30],[31]. We searched for significantly associated gene sets from Molecular Signatures Database subcatalog *C2*, a database of 1684 microarray experiment gene sets, pathways, and other groups of genes [31]. The analysis yielded many biologically informative sets (Dataset S2) consisting of genes enriched in brain cortex and heart atria, as well as biological processes such as mitochondrial fatty acid beta-oxidation, and reactive oxygen species. The application of GSA also identified 23 gene sets associated to genotoxic stress response (Table 1). *P53genes_all* is composed of transcriptional targets of *p53* (7157), a regulator of gene expression in response to various signals of genotoxic stress, with genes such as *GADD45A* (1647), *PMAIP1* (5366), and *SESN1* (27244) displaying repressed expression. *Genotoxins_all_24h_reg* consists of downregulated genes regulated in mouse lymphocytes at 24 hours by cisplatin, methyl methanesulfonate (MMS), mitomycin C, taxol, hydroxyurea, and etoposide [32]. Other gene sets consist of mostly downregulated genes in response to bleomycin, MMS, ultraviolet B (UVB), and ultraviolet C (UVC) radiation, which were also downregulated in the FRDA dataset (denoted by the negative GSA scores) (Table 1). We next asked if there were genes in common across the 23 genotoxic-stress-response gene sets. Transcripts present in at least 25% of the gene lists (81 genes total) were subjected to unsupervised clustering and displayed segregation into two groups of controls and patients (Figure 2A, right panels, and Dataset S3). Most of these genes fall in the gene ontology categories of transcription, signal transduction, and cell cycle (data not shown).

**Table 1:** Gene Set Analysis demonstrates a signature of DNA damage in FRDA patients.

Set Name	Score	*p*-Value	FDR
BLEO_HUMAN_LYMPH_HIGH_4HRS_UP	−0.37	0.044	0.6916
GENOTOXINS_ALL_24HRS_REG	−0.4663	0.044	0.6916
MMS_HUMAN_LYMPH_LOW_4HRS_DN	−1.1264	0.01	0.5726
MMS_MOUSE_LYMPH_HIGH_4HRS_UP	−0.7369	0.002	0.4453
P53GENES_ALL	0.3779	0.04	0.9698
UVB_NHEK1_C6	−0.4047	0.022	0.6203
UVB_NHEK1_DN	−0.4473	0.014	0.5726
UVB_NHEK2_DN	−0.3615	0.012	0.5726
UVB_NHEK3_C2	−0.7741	0.004	0.5726
UVC_HIGH_ALL_DN	−0.4171	0.006	0.5726
UVC_HIGH_D1_DN	−0.4286	0.032	0.668
UVC_HIGH_D2_DN	−0.366	0.064	0.7433
UVC_HIGH_D7_DN	−0.4576	0.018	0.5726
UVC_HIGH_D9_DN	−0.733	0.008	0.5726
UVC_TTD_4HR_DN	−0.5704	0.006	0.5726
UVC_TTD_8HR_DN	−0.3107	0.044	0.6916
UVC_TTD_ALL_DN	−0.4737	0.008	0.5726
UVC_TTD-XPCS_COMMON_DN	−0.4533	0.012	0.5726
UVC_XPCS_4HR_DN	−0.5225	0.014	0.5726
UVC_XPCS_4HR_UP	−0.6511	0.028	0.645
UVC_XPCS_8HR_DN	−0.5428	0.008	0.5726
UVC_XPCS_ALL_DN	−0.5414	0.006	0.5726
UV-CMV_UNIQUE_HCMV_6HRS_DN	−0.2488	0.02	0.5938

GSA was performed using the Molecular Signatures Database. Significantly enriched gene sets included 23 sets associated to genotoxic stress response.

To validate the gene expression changes observed in the child FRDA cohort, we examined an adult FRDA cohort (n = 14). These patients were compared with a new group of 15 adult controls, obtained from an NIEHS sponsored study [28] (Table S2). SAM analysis yielded 2,874 genes at an FDR of less than 0.018% (Dataset S4). This dataset was also analyzed with GSA, yielding significant gene sets related to genotoxic stress response, DNA repair, insulin response, and apoptosis (Dataset S5). When we performed unsupervised clustering of the same list of 81 genes found in the children with FRDA, we observed a similar segregation of patients from controls in this independent group of adults (Figure 2A), thus helping to validate this gene set.

The FRDA adult and FRDA children's datasets were further compared by limiting to significant SAM genes in common and taking into account the direction of differential expression. As stated earlier, a total of 1370 probesets were found significant in the children's cohort (FDR<0.023%), and the median FDR for these probesets in the second cohort was 0.34%. These analyses resulted in 228 mostly downregulated genes in common in both gene sets (FDR<0.018%; overlap *p* = 0.007 – based on the probability of the hypergeometric distribution) (Figure 2B). This list of 228 genes was analyzed for specific GO category enrichment (Figure 2C) (categories similar to those displayed in Figure 1 are not included; for the full list, see Table S3) and significantly associated to ubiquitin cycle, protein ubiquitination, and proteolysis/catabolism, as well as cell cycle. Using the Ingenuity Knowledge Base (see Materials and Methods), we further mapped the genes to biological function categories and specific pathways, which included apoptosis signaling and oxidative phosphorylation (Table S4). Additionally, the Fisher's exact test was used to search the Molecular Signatures Database for gene sets enriched with genes found in the overlap. These results yielded similar genotoxic gene sets as those observed with the children's data as a whole (Table S5).

### FRDA patients have significant mitochondrial and nuclear DNA lesions

Having seen a genotoxic stress response in the microarray data of the FRDA patients, we sought to validate these findings and test whether damage to the nuclear and mitochondrial genomes is elevated in patients with FRDA. Moreover, although genotoxic responsive gene sets in the children and adult cohorts had highly significant *p*-values, some had high false discovery rates, requiring further biological validation.

Blood DNA from the same 28 children studied for gene expression was analyzed using a quantitative PCR (QPCR) assay to detect DNA damage [33]. In addition to the 28 children evaluated by gene expression profiling, we obtained 19 more DNA samples from affected children in the same clinical study (Table S6) [22]. Only one 8.5 ml sample, per child, of whole blood was allotted for DNA isolation; all DNA samples were prepared by one person using the same protocol (see Materials and Methods). Blood from 15 young adults was obtained from an NIH blood bank in Bethesda, MD and used as controls (Table S6).

The QPCR technique we used has successfully identified lesions in nDNA and mtDNA, resulting from oxidative stress, in a number of organisms, including human and rat cell cultures [34]–[36], yeast [16], and mice [37]. However, this is the first time the assay has been used for DNA derived from human blood. The approach involves the amplification of a large 8.9 kb and 12.2 kb fragment for mtDNA and nDNA, respectively. Previous work by our group suggests that damage is distributed evenly throughout both genomes [34],[38],[39]. The large mtDNA amplification product constitutes ∼54% of the genome and is the representative of the overall genome. The primers used to amplify the product were designed to specifically avoid the D-loop region – a region that is often single-stranded and could increase DNA damage because of its high mutation frequency. Additionally, amplification of a short, ∼200 bp mtDNA fragment, which due to its small size has less chance of containing a lesion, is used to normalize for mitochondrial copy number in the amplification of the large mtDNA fragment. Oxidative damage induces a spectrum of lesions, such as strand breaks, abasic sites, and some base damage (i.e. thymine glycol), which interfere with the progression of the thermostable polymerase to replicate the template DNA. Thus, during amplification, the presence of an oxidative lesion results in the inability of the polymerase to synthesize the template DNA. The final amount of amplified DNA is inversely proportional to the number of oxidative lesions. The QPCR gene-specific damage assay is based on differential amplification of target genes in affected controls as compared to a control population. Excess DNA damage in the affected group will show as a decrease in amplification as compared to the control group.

**Figure 3:**
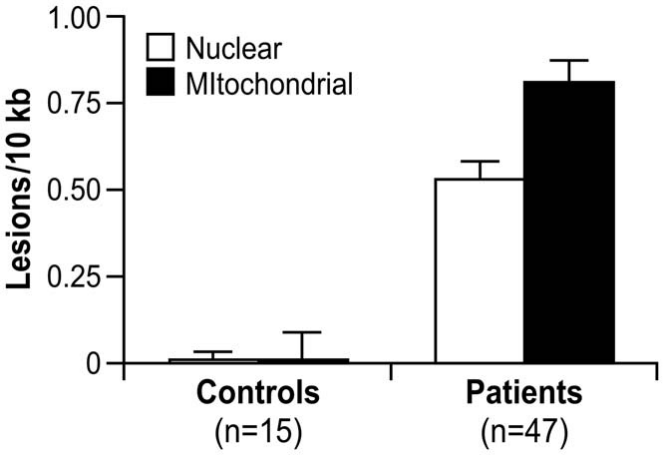
Nuclear and mitochondrial DNA damage are identified by QPCR analysis of blood DNA from 47 patients with FRDA and 15 controls. These data represent the number of excess lesions found per 10 kb of DNA from both mtDNA and nDNA genomes in FRDA patients as compared to the controls. A significant number of nuclear (0.53 lesions/10 kb) and mitochondrial DNA lesions (0.81 lesions/10 kb) were observed (*p*<0.0001, respectively, by Mann-Whitney U test). There is a significantly higher number of mitochondrial lesions than nuclear lesions (*p*<0.002 by Mann-Whitney U test), and both types of lesions are highly correlated (*p*<0.0001 by Spearman's Rank Correlation). Error bars represent the standard error of the mean.

A significant number of nuclear (0.53 lesions/10 kb) and mitochondrial DNA lesions (0.81 lesions/10 kb) was observed in the 47 FRDA children compared to 15 young adult controls (Table S6), with *p*<0.0001, respectively, by Mann Whitney U-test (Figure 3). There was also a significantly higher number of mitochondrial lesions than nuclear lesions (*p*<0.002, Mann Whitney U), and both mtDNA and nDNA lesions were found to be highly correlated by Spearman's rank test (*Rho* = 0.700; *p*<0.0001). Since this study did not have age-matched control children, for the two DNA damage variables (nDNA and mtDNA), we used a *t*-test to detect any association with age in the control group. This analysis was performed in the controls after stratifying the subjects as “higher” or “lower” than the median age of the group. Age was not found to be associated with mtDNA damage (*p* = 0.98) or nDNA damage (*p* = 0.10).

Finally, mtDNA and nDNA damage samples were classified as “high damage” or “low damage” if the lesions/10 kb of DNA was >0.85 or <0.85 (based on the distribution pattern; data not shown), respectively. GSA was then used to test the association of the DNA damage to predefined gene sets. A positive score would indicate enrichment in samples with high DNA lesions/10 kb, and a negative score would point to enrichment in samples with less lesions/10 kb. This analysis showed higher levels of DNA lesions associating to gene sets involving neuronal and synapse formation, as well as several others regarding a genotoxic stress response (*p*≤0.01) (Dataset S6).

### Extracting gene expression patterns identifies potential biomarkers of disease progression

**Figure 4:**
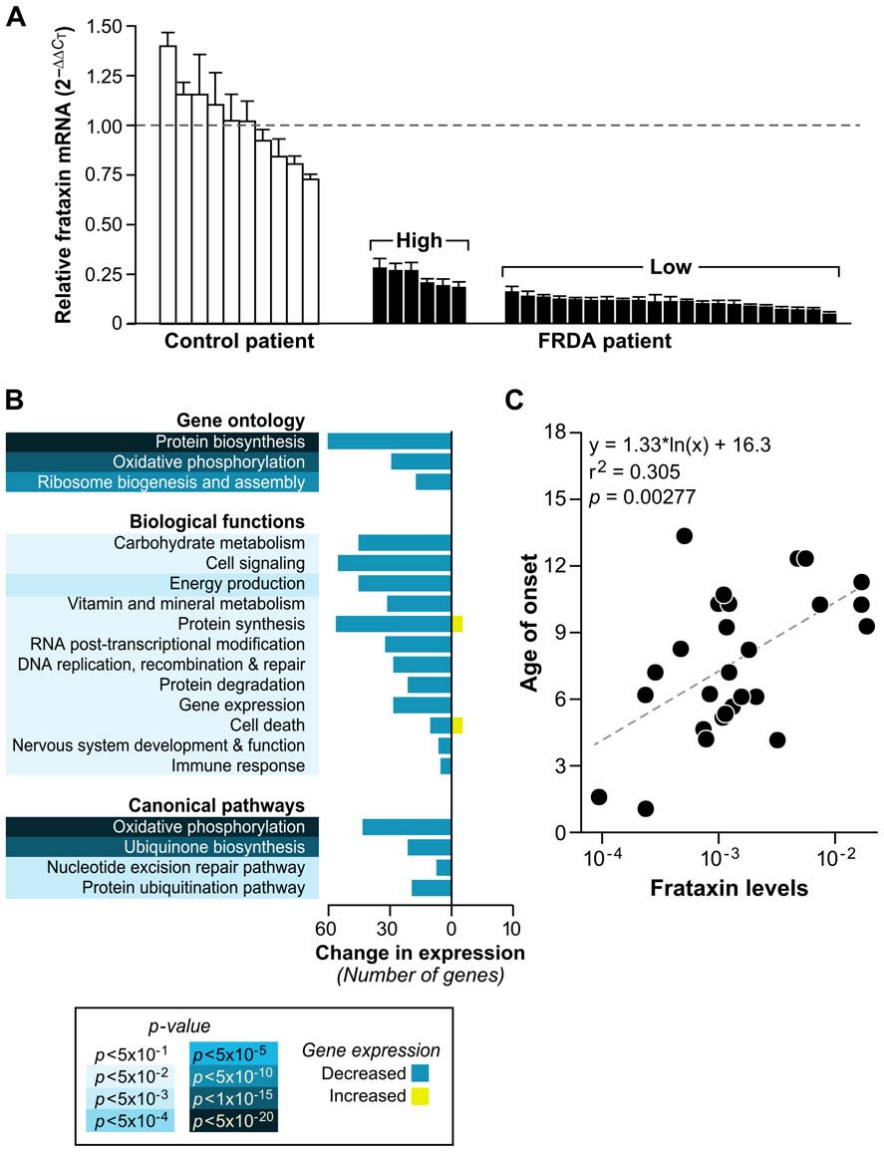
Patients with lower levels of frataxin correlate with age of onset of disease and have more compromised mitochondrial and protein biosynthetic function. (A) Real-time PCR of frataxin levels in all patients with available RNA (27) were compared to controls (10). Levels of frataxin are relative to the average Δ*C*_T_ of the controls (dotted line). The error bars represent standard deviations. Brackets encompass the patients stratified by their expression distribution into those expressing higher levels of frataxin (n = 6) and those expressing lower levels of frataxin (n = 21) (see Figure S1). (B) Global gene expression changes were analyzed in the patients with low levels of frataxin vs. patients with high levels of frataxin. Significantly differentially expressed genes (SAM FDR≤8%; *p*≤0.05; n = 973) were further examined for gene ontology groups categorized by biological process. The same list of genes was analyzed with IPA (Ingenuity Systems), which identified significant biological functions and canonical pathways. All gene groups contain mostly downregulated genes, indicating compromised mitochondrial and biosynthetic function in patients with the lowest expression of frataxin. (C) Age of onset plotted against the Real-time PCR frataxin levels yields a correlation of *r^2^* = 0.305 (*p* = 0.00277).

Since the bioinformatics analyses in this study were applied to SAM-derived data based on pooling the raw data from all 28 children (Materials and Methods), we tested the hypothesis that a discrete set of genes would be differentially expressed in patients with the lowest levels of frataxin. We next generated a transcription profile comparing patients with the lowest amount of frataxin, analyzed by Real-time PCR, to those with the highest. The samples were stratified into two groups, based on the expression distribution, where the values formed two distinct modes (see Figure S1 legend). A threshold of −2.5 was selected to separate these two modes, resulting in six patients considered “high-frataxin expressers” and 21 patients designated “low-frataxin expressers” (Figure 4A and Figure S1). Significant gene changes were determined using SAM at a cutoff of FDR≤8% (*p*≤0.05) for a total of 973 genes. These genes were analyzed for gene ontology enrichment and mapped to pathways in the Ingenuity Knowledge Base. Top scoring categories and pathways included protein biosynthesis, oxidative phosphorylation, ubiquinone biosynthesis, nucleotide excision repair, and protein ubiquitination, all of which were downregulated in those patients expressing lower levels of frataxin (Figure 4B).

Since we had quantified the level of frataxin for each patient, we sought to find an association of these levels to the expression of each gene in our genotoxic signaling list (Dataset S3). A univariate linear model was constructed to test this association, and no genes in this set were found to be significantly associated with frataxin levels. The minimum *p*-value was 0.002, which was not significant after multiple testing correction (*q* = 0.20). We further sought to find correlations of frataxin mRNA levels to all available clinical data for each child, including DNA lesions and disease duration, but only found an association, by univariate linear modeling, with age of onset and number of short GAA repeats. This resulted in *p* = 0.00277 and *r^2^* = 0.305 for the relationship of frataxin mRNA levels to age of onset (Figure 4C), and *p* = 0.00131 (*r^2^* = 0.344) for the relationship of frataxin mRNA levels with the short GAA repeat length (Figure S2). These correlations of frataxin mRNA to short GAA repeat length and age of onset are in agreement with other published studies [1],[40],[41].

Intrigued by these associations to clinical data, we wondered if we could directly associate the global gene expression data to all clinical data and decided to use a method called EPIG, which **e**xtracts microarray gene expression **p**atterns and **i**dentifies co-expressed **g**enes (see Materials and Methods) [42]. Indeed, not only did we validate the SAM gene lists for the FRDA children, but we extracted patterns that associated the mostly downregulated transcriptional changes to their frataxin levels, age of diagnosis, and International Cooperative Ataxia Rating Scale scores – a scoring method used to discern the level of disability in the FRDA patient (Table 2) [43]. Patterns correlating levels of frataxin to gene expression encompassed 37 responsive genes. These genes were grouped most strongly in the GO categories of immune response and protein biosynthesis. Patient age of diagnosis and ICARS score associated with 98 and 48 genes, respectively, with protein biosynthesis as a highly significant category. Supervised correlation analysis was also performed, where correlation *r* values and *p*-values between each gene expression profile and ICARS score were calculated. This analysis generated a list of 144 positively and negatively correlated genes at a *p*-value threshold of 0.01. Protein biosynthetic function was once again highly significant as a GO category, resulting from the input of these genes (Table 2). Genes correlated with ICARS are potential biomarker candidates that may help to classify the progression of the disease.

**Table 2:** Potential biomarkers of FRDA: gene associations to clinical data based on differential expression.

Clinical Data	Gene Category	*p*-Value	Gene Symbol
*Frataxin Levels*	n = 37		
	Immune Response (*BP*)	9.79E-06	CXCL11; FPR1; HLA-C; IFITM1; LILRB3; POU2F2; S100A12; S100A9; S100B; TLR4
	Protein Biosynthesis (*BP*)	6.14E-04	RPS20; RPS23; RPS26; RPS27A; RPS4X; S100B; TLR4
	Inflammatory Response (*BP*)	7.55E-04	CXCL11; FPR1; S100A12; S100A9; TLR4
	Eukaryotic 48s Initiation Complex (*CC*)	3.83E-05	RPS20; RPS23; RPS26; RPS4X
	Ribosome (*CC*)	2.35E-03	RPS20; RPS23; RPS26; RPS27A; RPS4X
*Age of Diagnosis*	n = 98		
	Protein Biosynthesis (*BP*)	1.83E-02	MAN1B1; RPL37; RPL3L; RPS20; RPS23; RPS4X; RPS5
	Cytosolic Ribosome (*CC*)	2.64E-04	RPL37; RPS20; RPS23; RPS4X; RPS5
*ICARS^1^*	n = 49		
	Protein Biosynthesis (*BP*)	2.74E-05	MRPS14; RPL24; RPL26L1; RPL27; RPL37; RPS19; RPS23; RPS27A; RPS3
	Ribosome (*CC*)	3.04E-10	HAT1; MRPS14; MRPS28; RPL24; RPL26L1; RPL27; RPL37; RPS19; RPS23; RPS27A; RPS3
*ICARS^2^*	n = 144		
	Protein Biosynthesis (*BP*)	1.41E-11	FAU; GSPT2; NHP2L1; RPL13A; RPL18A; RPL19; RPL22; RPL23A; RPL28; RPL29; RPL36; RPL37; RPL37A; RPLP2; RPS10; RPS12; RPS13; RPS14; RPS15; RPS19; RPS23; RPS28; RPS3
	Ribosome (*CC*)	3.78E-19	FAU; MTHFR; NHP2L1; ONECUT1; RPL13A; RPL18A; RPL19; RPL22; RPL23A; RPL28; RPL29; RPL36; RPL37; RPL37A; RPLP2; RPS10; RPS12; RPS13; RPS14; RPS15; RPS19; RPS23; RPS28; RPS3
	Cytoplasm (*CC*)	8.50E-08	ACTN4; ADSS; AP2B1; APRT; ATP5G2; CCND3; CDC42BPB; CES1; CFL1; CRIP2; CTSF; DSG1; FAU; FHL2; GLI2; HIPK2; HSPC152; ITGAE; KIAA0907; LOC51334; MAP4; MTHFR; NGFRAP1; NHP2L1; NME3; NXT1; ONECUT1; PAPSS2; PEX6; RPL13A; RPL18A; RPL19; RPL22; RPL23A; RPL28; RPL29; RPL36; RPL37; RPL37A; RPLP2; RPS10; RPS12; RPS13; RPS14; RPS15; RPS19; RPS23; RPS28; RPS3; SLC27A5; STX6; TMEM9; UXT; XPO7
	Structural Constituent of Ribosome (*MF*)	2.06E-19	FAU; MRPS24; NHP2L1; RPL13A; RPL18A; RPL19; RPL22; RPL23A; RPL28; RPL29; RPL36; RPL37; RPL37A; RPLP2; RPS10; RPS12; RPS13; RPS14; RPS15; RPS19; RPS23; RPS28; RPS3

n = significant genes; selected categories and their genes are displayed.

Frataxin levels, Age of Diagnosis, ICARS^1^; n = genes found by EPIG analysis.

ICARS^2^; n = genes found by supervised correlation analysis, where the *r* and *P* values between gene expression and ICARS score were calculated.

BP = Gene ontology system, biological process.

CC = Gene ontology system, cellular component.

MF = Gene ontology system, molecular function.

### Analysis of the second patient cohort shows consistent alterations in gene expression with FRDA children

EPIG was further utilized to extract patterns and compare between the FRDA children and FRDA adults. We were interested in finding biomarker gene candidates based on duration of disease and representative of severity. Of particular interest were genes that were very significant in both cohorts, regulated in the same direction, but displaying larger differential expression in the adults. A detailed examination of the gene profiles was employed using the signal to noise ratio, ANOVA, Student's *t*-test, and fold changes, and resulted in fifteen genes that are potential biomarkers of disease progression in need of further testing: *SERPINC1* (462), *DHFRL1* (200895), *IRX2* (153572), *SGCE* (8910), *ADAM23* (8745), *TBX3* (6926), *SLC5A4* (6527), *CCAR1* (55749), *MS4A2* (2206), *IMPACT* (55364), *NDUFA5* (4698), *CEACAM6* (4680), *C10orf88* (80007), *ITGA4* (3676), and *CD69* (969) (first seven genes are upregulated and the subsequent eight genes are downregulated). Entrez IDs for these genes and all genes described in this paper can be found in Table S7.

## Discussion

**Figure 5:**
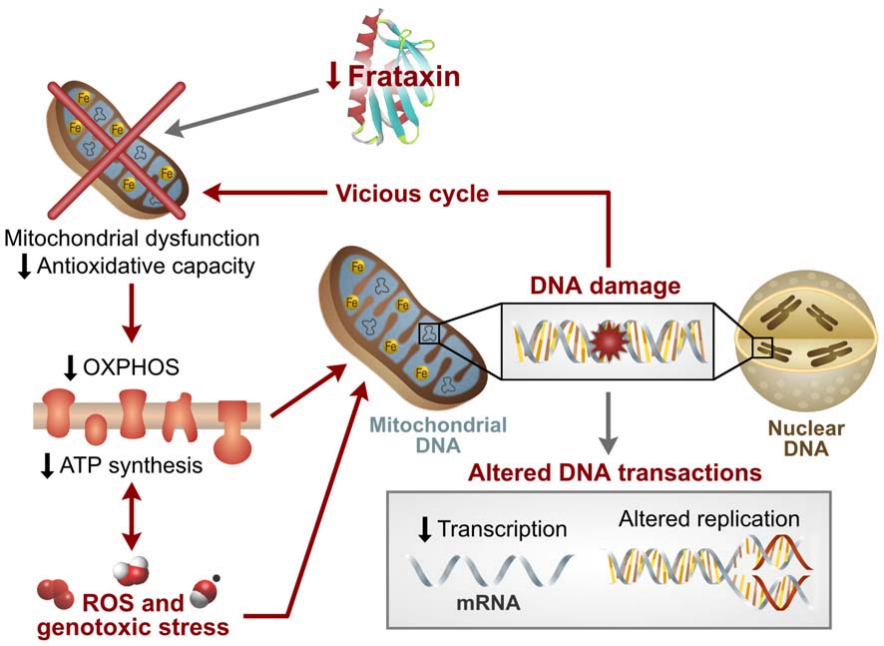
Model of Friedreich's ataxia pathology based on this study. Data presented in this study are consistent with a dysregulation of mitochondrial function, decreased oxidative phosphorylation, increased ROS production, and subsequent mitochondrial and nuclear DNA damage. These factors contribute to decreased signaling and altered DNA transactions, which are likely to result in subsequent loss of protein synthesis and decreased protein degradation, as suggested in the transcription profiling. These alterations may cause tissue damage, altered immune response, and the clinical pathology associated with FRDA.

This study provides the first summary of gene expression changes in the blood of 28 children with Friedreich's ataxia and the association of this global response to the nuclear and mitochondrial DNA damage found in 47 children (including the 28 children from the gene expression analysis). These data – the 23 gene sets associated to a genotoxic stress response and the direct biological evidence of mtDNA and nDNA damage in the blood – result in a working model of the disease, where repressed levels of frataxin create a vicious cycle of mitochondrial dysfunction (probably due to ISC biosynthesis impairment), decreased oxidative phosphorylation, and increased reactive oxygen species production and genotoxic stress (Figure 5). These events result in DNA damage and altered DNA transactions, which likely contribute to the decreased protein biosynthesis, signaling, transcription, DNA replication/recombination/repair, apoptosis, and ubiquitination, as well as the altered immune response and proliferation indicated in the transcription profiling. Such changes in the blood may relate to the clinical manifestations of neurodegenerative and cardiovascular disease in FRDA patients, while also providing biomarker candidates for their disease.

### Gene expression changes in peripheral blood of FRDA patients compared to lymphoblastoid cell lines

Cortopassi and coworkers have previously published two global gene expression analyses of cells from FRDA patients [25] and tissue from frataxin-KO mice (26). While direct analyses of these published studies with the work presented here is not possible, since these previous data were not apparently deposited in a public database, several points are worth noting. Tan et al. [25] reported 48 significant frataxin-dependent differentially expressed genes in at least two of the human cell types. In particular, they focused on seven downregulated transcripts belonging to the sulfur amino acid (SAA) and iron-sulfur cluster biosynthetic pathways. When they combined data from mouse and human frataxin-deficient cells and tissues, mitochondrial coproporphyrinogen oxidase (*CPOX*; 1371), which is involved in the heme pathway, and the homologue of yeast *COX23* (856516) were most consistently downregulated [27]. These transcripts were not found to be significant in our dataset. The authors conclude that frataxin deficiency leads to heme deficiency. While our work with both FRDA patient cohorts did not show the heme pathway as significantly repressed, the downregulated mitochondrial pathways we did observe are easily affected by, or could contribute to, heme deficiency. Ultimately, these changes in heme biosynthesis could cause DNA damage [44] recapitulated in our patients.

We wanted to further validate the datasets derived from children and adults with FRDA, so we analyzed ten FRDA lymphoblastoid cell lines, compared with seven age-matched controls, and data from a previous report involving two lymphoblastoid cell lines (one control and one affected) (Table S8) [45]. Particularly interesting overlaps with the blood data were observed with GSA analysis, which also yielded significant gene sets related to genotoxic stress response (for biologically informative sets in common between the FRDA children's data and the lymphoblastoid data, see Dataset S7 and Figure S3). Furthermore, GSA analysis of the lymphoblastoid data also found an association to significant gene sets like *electron_transport_chain*, *mitochondrion*, and *ubiquinone biosynthesis*, which are indicative of the mitochondrial dysfunction expected in frataxin-deficient cells (Dataset S7).

### Consequences of mitochondrial and nuclear DNA damage in FRDA patients

While the QPCR assay used in this study cannot directly identify the type of DNA damage inhibiting the progression of the thermostable polymerase, the increase in mtDNA damage, as compared to nDNA damage, is consistent with a large number of studies from our and other laboratories, indicating that mtDNA is more prone to oxidative stress [16], [34]–[37]. The increased DNA damage observed in the children suggests oxidant injury in their blood cells, probably caused by an increase in bioavailable iron in the mitochondria. Persistent mtDNA damage in FRDA patients could impair mitochondrial function. Experiments with mammalian cell cultures, treated with hydrogen peroxide, indicate that relentless mtDNA damage decreases oxidative phosphorylation and ATP production (unpublished observation). *In vivo* evidence of impaired mitochondrial ATP production has, in fact, been seen in the muscle of FRDA patients [46],[47] and in KO mice [48]. Karthikeyan et al. [16] also demonstrate how yeast strains with reduced frataxin accumulate mitochondrial iron and generate reactive hydroxyl radicals, which damage cell membranes, proteins, and mitochondrial DNA, resulting in the decreased capacity for ATP synthesis through impaired oxidative phosphorylation. The same study further demonstrates how low levels of frataxin in a *RAD52* double-strand break repair deficient yeast strain lead to rapid G2/M cell cycle arrest, which is consistent with nuclear damage. Moreover, reports depicting an increased sensitivity to gamma irradiation in FRDA skin fibroblasts, and the induction of chromosomal damage by mutagens in blood lymphocytes, support a hypothesis of increased susceptibility and/or altered DNA repair capacity in these patients [49],[50].

The eukaryotic cell, in response to DNA damage, employs different strategies for damage recognition and repair in order to maintain the integrity of the genome. DNA damage sensors such as *ATM* (472) and *p53* are crucial in detecting double-strand breaks and general DNA damage responses, respectively [51]. Although the *ATM* and *p53* genes are not differentially expressed in the datasets presented here, we observed, via GSA and the Ingenuity Knowledge Base (data not shown), that many interacting network partners of these two proteins are significantly altered.

The significant increase in persistent DNA damage we found in FRDA patients, as well as the transcription profiling results, indicate altered repair capacity and altered apoptosis signaling events. Furthermore, a significant number of genes included in the Ingenuity biological function category of Cancer (104 and 475 genes in Cancer and its subcategories, *p*≤0.05, for FRDA children and FRDA adults, respectively; data not shown) may explain the malignant transformation potential of frataxin-deficient cells, both *in vitro* and *in vivo* [10],[48],[52]. This hypothesis is supported by work done in mice by Thierbach et al. [48], albeit a mouse model completely deleted for frataxin. When they disrupted expression of frataxin in mouse hepatocytes, lifespan was not only reduced, but the livers had increased oxidative stress and mitochondrial dysfunction. This was paralleled by reduced activity of iron-sulfur cluster containing proteins and the development of multiple hepatic tumors. The authors also reported impaired phosphorylation of the stress-inducible p38 MAP kinase and suggest that frataxin may, in fact, be a mitochondrial tumor suppressor protein. Thus, while reports of cancer in FRDA patients are rare [53]–[56], the incidence may be underestimated due to premature mortality of these patients in early adulthood.

The overall decrease in transcription and DNA damage we observed are likely consequences of dysfunctional ISC biosynthesis and reduced activity of proteins containing iron-sulfur centers. In fact, several damage recognition and DNA repair proteins are iron-sulfur containing and could be directly linked to the DNA damage described [57]–[60]. Currently, we are analyzing protein levels, in frataxin-deficient cell lines, of a panel of iron-sulfur containing proteins important to DNA repair. Some candidate proteins with iron-sulfur centers include the MutY (4595) homologue (a glycosylase in base excision repair); the yeast protein, Rad3 (856918), which is essential for viability, and its human homologues XPD (2068) and Fancj (83990) (helicases involved in nucleotide excision repair and the Fanconi anemia repair pathway, respectively); and Pri2 (853821) (essential to RNA primer synthesis) [57]–[60].

The likely sequence of events leading to the DNA damage we observed are as follows: 1) deficiency of frataxin generates a defect in ISC assembly and biogenesis [5], [12]–[14]; 2) the dysfunction of biosynthesis of mitochondrial iron-sulfur clusters, and deficient ISC enzyme activity, produces a defect in heme and a lack of cytochrome C [12]; 3) impairment of electron transport activity, which is dependent on iron-sulfur biogenesis, and the decrease in cytochrome C, results in higher levels of ROS production [12],[14]; and 4) due to lack in antioxidative capacity, which we explain below, eventual DNA damage occurs. Therefore, we believe the DNA damage in the blood of the Friedreich's ataxia patient is a secondary event to the primary one of frataxin depletion and neurodegeneration. However, the secondary event of cellular oxidative stress and DNA damage is a significant component to the underlying pathology of the disease.

### Adaptation to chronic stress

We further propose that the downregulation of many key pathways (Figure S4A and Figure 4C) and GO categories (Figure 1, Figure 2C, and Figure 4B), in this study, may suggest a systemic survival response to chronic genotoxic stress and consequent DNA damage. Chronic genotoxic stress in FRDA probably results from iron accumulation in the mitochondria, and it might be expected that cellular redox homeostasis, such as that regulated by *NRF2* (4780), would protect the cell from excessive reactive oxygen metabolites. However, we observed the downregulation of the *NRF2*-mediated oxidative stress pathway (Figure S4B), strengthening published reports suggesting a disabled antioxidant defense response in FRDA [9],[10],[61], including a recent study by Paupe et al. [62] showing that cultured fibroblasts from patients with FRDA exhibit hypersensitivity to oxidative stress because of an impaired *NRF2* signaling pathway. Furthermore, Chantrel-Groussard et al. [9] found that reduced frataxin does not induce superoxide dismutases nor the import iron machinery by endogenous oxidative stress in FRDA fibroblasts compared to controls. Superoxide dismutase activity is also not induced in the heart of conditional knock-out mice [21]. Conversely, overexpression of human frataxin in murine cells increases antioxidant defense via activation of glutathione peroxidase and elevation of reduced thiols, and reduces the incidence of ROS-induced malignant transformation [10]. Sturm et al. [61] reported data strongly indicating that a reduction in frataxin does not affect the mitochondrial labile iron pool in human cell lines and suggests that these cells have a decreased antioxidative capacity. Overall, these studies support a mechanism by which iron-sulfur proteins are lost [13] and there are increased amounts of ROS and a disabled antioxidant defense system.

Based on our blood analysis of FDRA patients showing chronic genotoxic stress responses and chronic DNA damage, we believe these stressors cause a genetic reprogramming of fundamental biological pathways as a protective survival response. A similar idea was reported by Niedernhofer et al., [63] who analyzed a case of *XPF/ERCC1* (2072/2067) progeroid syndrome and a knockout mouse model of this disease. They concluded that chronic DNA damage causes cells to deemphasize growth activities in order to ensure organismal preservation and maximal lifespan, despite an increase in cellular senescence and apoptosis.

The level of injury in the cells of these patients is not only exacerbated by the loss of antioxidative defense, but also by the downregulation of oxidative phosphorylation and the shutdown of protein synthesis and translation, as was observed in the gene expression analysis of patients with lower levels of frataxin as compared to patients with higher levels. EPIG analysis further demonstrated a marked decrease in genes involved in protein synthesis, and genes encoding ribosomal proteins, correlating with frataxin levels, age of diagnosis, and ICARS scores. Many of the significant genes involved in the category of protein synthesis include the repression of several initiation factors. Paschen et al. [64] discuss how such events suggest the relationship between the shutdown of translation and induction of neuronal cell death. It is our hypothesis that such global responses are triggered by chronic stress.

We also found interesting the effect frataxin deficiency has on ubiquitin cycle and protein degradation in both FRDA children and FRDA adults. Modifications to the function of ubiquitinating enzymes by oxidative stress have been reported [4]. Degradation of damaged proteins by the ubiquitin-proteasome system (UPS) is one of the most important processes in the cell, and a decreased capacity for protein degradation is related to several neurodegenerative diseases and pathologies of the inflammatory immune response [65],[66].

In summary, this study provides the first evidence of increased mitochondrial and nuclear DNA damage, as well as gene expression patterns consistent with DNA damage, in peripheral blood cells of patients with FRDA. Analyses of clinical features and gene expression patterns correlate with age of onset and frataxin mRNA levels, as well as altered protein synthesis with frataxin levels, ICARS score, and age of diagnosis. Future studies with Friedreich's ataxia patients will help better define these gene sets and DNA damage as candidate biomarkers of disease severity and progression. Additionally, biomarkers are vital to the development of therapeutic approaches, and our study points to possible drug interventions, like modulating the ubiquitin-proteasome system or upregulating molecular chaperone activity, which may be as useful for FRDA as they are in other neurodegenerative diseases. However, the development of effective therapeutic approaches also depends on an enhanced understanding of signaling pathways and other cellular responses to chronic genotoxic stress.

## Materials and Methods

### Ethics statement

Peripheral blood samples were collected from 48 children with FRDA participating in a randomized, placebo-controlled clinical trial for idebenone [registered with ClinicalTrials.gov, number NCT00229632, and approved by the NIH Institutional Review Board at the National Institute of Neurological Disorders and Stroke (NINDS), protocol # 05-N-0245]. Samples from 14 anonymous FRDA adults were collected in the reference center clinic dedicated to cerebellar ataxias and aspartic paraplegias at the University Salpêtrière Hospital in Paris; samples were exempted by the NIH Institutional Review Board at the National Institute of Environmental Health Sciences (NIEHS), exempt # 3984. All controls used for transcriptional profiling were young healthy adults from an acetaminophen study [28], approved by the Institutional Review Board at the University of North Carolina, Chapel Hill, protocol # GCRC-2265. Controls used for the DNA damage assay were obtained from an NIH blood bank. Blood and/or apheresis samples were obtained from healthy volunteer donors who gave signed consent to participate in an IRB-approved protocol for use of their blood in laboratory research studies; these samples were approved by the Institutional Review Board at the National Cancer Institute (NCI), protocol # 99-CC-0168.

### RNA isolation from blood

Peripheral blood samples were collected from 48 children with FRDA participating in a randomized, placebo-controlled clinical trial. All whole blood samples in this study were collected before administration of idebenone. A detailed description of all subjects and clinical endpoints was recently published [22]. Due to other endpoints, this study only allotted one 8.5 ml sample of blood from each patient for RNA isolation. RNA was isolated by one person, utilizing the PAXgene blood RNA isolation kit (PreAnalytiX/QIAGEN, Hilden, Germany) according to the manufacturer's protocol, including the optional on-column DNase digestion, except that the centrifugation time after proteinase K digestion was increased from 3 to 20 minutes in order to obtain a tighter debris pellet. RNA quality was assessed with an Agilent Bioanalyzer (Palo Alto, CA) to ensure that samples with intact 18S and 28S ribosomal RNA peaks were used for microarray analysis. Of the 48 patients, twenty samples were lost during the isolation procedures, leaving 28 high-quality RNA samples remaining. The demographics for these subjects are detailed in Table S1. RNA was also isolated, using the same methods already described, from 14 anonymous FRDA adults (Table S2). All controls used were young healthy adults (see demographics data in Table S1 and Table S2) from an acetaminophen study [28]. Two independent sets of control populations were used separately to compare to the children and the adult validation cohort.

### Gene profiling

Gene expression profiling was conducted using Agilent Human 1A(V2) Oligo arrays with ∼20,000 genes represented (Agilent Technologies, Palo Alto, CA). Each sample was hybridized against a human universal RNA control (Stratagene, La Jolla, CA). 500 ng of total RNA was amplified and labeled using the Agilent Low RNA Input Fluorescent Linear Amplification Kit, according to manufacturer's protocol. For each two color comparison, 750 ng of each *Cy3*- (universal control) and *Cy5*-labeled (sample) cRNA were mixed and fragmented using the Agilent *In Situ* Hybridization Kit protocol. Hybridizations were performed for 17 hours in a rotating hybridization oven according to the Agilent 60-mer oligo microarray processing protocol prior to washing and scanning with an Agilent Scanner (Agilent Technologies, Wilmington, DE). The data were obtained with the Agilent Feature Extraction software (v9.1), using defaults for all parameters. The Feature Extraction Software performs error modeling before data are loaded into a database system. Images and GEML files, including error and *p*-values, were exported from the Agilent Feature Extraction software and deposited into Rosetta Resolver (version 5.0, build 5.0.0.2.48) (Rosetta Biosoftware, Kirkland, WA). All gene expression data have been deposited in the public Gene Expression Omnibus (GEO) database and are available under the series ID GSE11204.

### Statistical and data analyses

Supervised analysis to find genes associated with case versus control or low frataxin expression versus high expression was performed using Significance Analysis of Microarrays (SAM) after pooling the raw data [29]. The two-class unpaired SAM algorithm was used and the false discovery rate was set to less than or equal to 1% for all analyses. Gene Set Analysis (GSA) [30] was also performed for these comparisons to test the association of gene sets instead of individual genes. The database of gene sets used for GSA was obtained from the Molecular Signatures Database (MSigDb) [30]. Gene sets demonstrating a *p*-value less than 0.01 were considered significant.

Biologically relevant themes in the lists of significant genes from SAM were analyzed with gene ontology tools, GoMiner and DAVID (Database for Annotation, Visualization and Integrated Discovery) [67],[68]. GO terms with *p*≤0.05 for upregulated, downregulated, and/or combined direction of change were selected for analysis. Both tools group genes according to the GO categories of biological process, cellular component, and molecular function, based on ranking by a hypergeometric test *p*-value. These data were also uploaded into Ingenuity Pathway Analysis (IPA) software v 5.5.1 (Ingenuity Systems, Redwood City, CA), a program that categorizes genes into biological functions but also enables visualization of biologically relevant networks and canonical pathways (“canonical” implies “established”). Go to www.Ingenuity.com for specifics regarding the application. Unsupervised clustering and heat-map generation were carried out with Cluster and Treeview programs [69].

The levels of DNA damage were analyzed by Mann-Whitney U test or Spearman's Rank Correlation because the data is not normally distributed or homoskedastic.

In order to test association between gene expression and age, we used SAM. For the two DNA damage variables we used a Student's *t*-test to detect association with age. All these analyses were performed in the controls, and age was dichotomized by comparison to the median age of the controls.

A univariate linear model was constructed to test the association of each gene in the genotoxic gene set list (genes found in 25% of gene lists from significant gene sets) to patient frataxin levels (determined by Real-time PCR). Correlations of frataxin mRNA levels to all available clinical data for each child were also performed by univariate linear modeling.

In extracting gene expression patterns, EPIG [42] uses a filtering process where all profiles initially are considered as pattern candidates. Briefly, using all pair-wise correlations, any candidate profile, whose local cluster size is less than a predefined size M*_t_* or its correlation with another profile is higher (>R*_t_*) but has a lower local cluster size M, is removed from pattern construction consideration. Among the remaining profiles, EPIG then creates representative profiles for the corresponding local clusters and removes those profiles with a signal-to-noise ratio or magnitude less than given thresholds. After this filtering processing, the remaining profiles consist of the extracted patterns, which are used to be the representatives to each of the local clusters. Subsequently, EPIG categorizes each significant gene to a pattern, for which it has the highest correlation with the gene profile. A gene not assigned to any extracted pattern is considered an “orphan” if its highest correlation *r*-value is lower than the given threshold R.

### TaqMan real-time PCR

The frataxin probe on the Agilent chip was observed to lack sensitivity for both the individual lymphoblastoid cell lines from affected people (data not shown) and the whole blood from FRDA patients. We, therefore, decided to obtain relative gene expression levels of frataxin by TaqMan Real-time PCR [70]. The sequence information of the probe used for TaqMan is the proprietary information of PE Applied Biosystems, but we know it is 75 nucleotides that span across the exon 1 and exon 2 boundary. On the other hand, the probe on the microarray chip is mostly from the 3′ UTR of frataxin.

One microgram of total RNA from 27 patients and 10 controls was used for reverse transcription with TaqMan Reverse Transcription Reagents (PE Applied Biosystems). To determine relative frataxin mRNA levels, Real-time PCR was carried out using the ABI Prism 7900HT sequence detection system. Primer and probe sets for frataxin and glucuronidase-beta were purchased as pre-developed assays from PE Applied Biosystems. Relative quantification was obtained using the threshold cycle method after verification of primer performance, following the manufacturer's guidelines. The levels of frataxin obtained are relative to the average Δ*C*_T_ from 10 controls.

### Mitochondrial and nuclear DNA quantitative PCR assay

Total DNA from whole blood was successfully isolated from 47 children enrolled in the study and 15 adult controls obtained from an NIH blood bank in Bethesda, MD, (Table S6) using the PAXgene blood DNA isolation kit (PreAnalytiX/QIAGEN, Hilden, Germany) according to the instructions of the manufacturer. Briefly, one 8.5 ml sample of whole blood, per child, was collected in PAXgene blood DNA tubes for DNA isolation; each blood sample was transferred to a processing tube containing a lysing solution. Lysed red and white blood cells were centrifuged, and the resulting pellet of nuclei and mitochondria was washed and resuspended. After digestion with protease, DNA was precipitated by addition of isopropanol and dissolved in water. All DNA samples were prepared by one person. DNA lesion frequencies were calculated as described previously [33]. Briefly, the amplification of patient samples (*A_patient_*) was compared to the amplification of non-damaged controls (*A_control_*) resulting in a relative amplification ratio. Assuming a random distribution of lesions and using the Poisson equation [*f*(*x*) = *.e* ˜^−λ^ λ^x^/*x*!, where λ is the average lesion frequency for the nondamaged template (i.e., the zero class; *x* = 0)], the average lesion per DNA strand was determined by the following equation: .λ = .−ln *A_Patient_*/*A_control_*. Amplification of the large mitochondrial target was normalized to mitochondrial copy number by examination of a short mitochondrial target, which due to its short size, should be free of damage.

## Supporting Information

Figure S1 Stratification of Friedreich's ataxia patients based on the distribution of frataxin expression levels. A density plot of frataxin (FXN) expression by Real-time PCR illustrates the distribution of FXN expression over the cases. The x-axis indicates the change in cycle threshold (ddCT) in the cases relative to a pool of the controls. A threshold of −2.5 was selected to split the cases into those with high expression of FXN (6 cases) versus those with relatively low expression of FXN (21 cases).
(0.17 MB TIF)

Figure S2 GAA repeat length correlates with frataxin levels. Individual FXN levels were determined for each patient by real-time PCR. A univariate linear model was constructed to test the association of frataxin mRNA levels with short GAA repeats. The short GAA repeat length correlated with mRNA levels (*r* ^2^ = 0.338, *p* = 0.00131).
(0.17 MB TIF)

Figure S3 Gene Set Analysis finds gene sets in common between the lymphoblastoid cell line and the FRDA children datasets. Significantly associated gene sets from Gene Set Enrichment Analysis subcatalog *C2*, a database of 1,684 microarray experiment gene sets, pathways, and other groups of genes, were identified for both the lymphoblastoid cell line and FRDA children datasets. The analysis yielded many biologically informative sets (n = 171, *p*≤0.05 and n = 120, *p*≤0.05 for the lymphoblastoids and FRDA children, respectively) with 37 gene sets in common for the two datasets. The Venn diagram displays 8 selected gene sets that associate with both datasets. Descriptions in black loosely summarize the gene set's association to phenotype, cells, tissue, or pathway as described by the authors of origin in the database.
(0.99 MB TIF)

Figure S4 Highly significant pathways responding to frataxin reduction in FRDA patients are driven by downregulation. Significant *p*-values were calculated by the right-tailed Fisher's Exact test using the entire dataset in the Ingenuity Pathway Analysis program. (A) Significance of the top signaling and metabolic pathways (*p*-value≥0.05) in the complete list of differentially expressed genes compared to that of downregulated genes only (FDR<0.023%). Upregulated genes only did not reach significance and were not included. (B) Frataxin deficiency downregulates genes involved in the *NRF2*-mediated oxidative stress pathway (adapted from the Ingenuity Pathway Analysis Knowledge Base). Induced and repressed genes are depicted in yellow and blue, respectively. Genes with no borders are significant in FRDA children, genes with black borders are significant in FRDA adults, and genes with red borders are significant in both. Significant genes in FRDA children not shown: *FKBP5* (↓). Significant genes in FRDA adults not shown: *ASK1* (↓), *MEK5* (↓), *JNK1/2* (↓), *EIF2AK3* (↓), *GSTO1* (↓), *GSTA1* (↑), *GSTM3* (↑), *JUN* (↑), *MAFG* (↓), *NQO1* (↑).
(3.92 MB TIF)

Table S1 Demographics for Friedreich's ataxia children involved in gene expression analysis of peripheral blood.
(0.03 MB DOC)

Table S2 Demographics for Friedreich's ataxia adult subjects involved in gene expression analysis of peripheral blood.
(0.03 MB DOC)

Table S3 The complete list of GO terms associated to the 228 significant genes in common between the FRDA children and FRDA adults.
(0.06 MB DOC)

Table S4 Ingenuity Pathway Analysis. Ingenuity Knowledge Base biological function categories and pathways associated to the overlap of 228 significant genes from the FRDA children and FRDA adults, respectively (*p*≥0.05).
(0.11 MB RTF)

Table S5 Gene Set Analysis of common genes. Enriched gene sets associated to significant genes (SAM) that overlap between FRDA children and FRDA adults.
(0.04 MB DOC)

Table S6 Demographics for Friedreich's ataxia children involved in DNA damage analysis of peripheral blood.
(0.03 MB DOC)

Table S7 Gene names and corresponding IDs.
(0.19 MB RTF)

Table S8 Demographic data for FRDA and control lymphoblastoid cell lines. Data from a previous lymphoblastoid gene expression analysis [45] was of limited use due to replicate noise, difference in microarray platform (they used Affymetrix), and only one affected and one control lymphoblastoid comparison; thus, few comparisons could be drawn between our lymphoblastoid data or our cohort data with their data.
(0.07 MB RTF)

Dataset S1 SAM list of genes for the children's cohort.
(4.44 MB XLS)

Dataset S2 Biologically informative gene sets for the FRDA children using Gene Set Analysis.
(0.16 MB XLS)

Dataset S3 Transcripts present in at least 25% of the gene lists from genotoxic stress gene sets found with Gene Set Analysis.
(0.03 MB XLS)

Dataset S4 SAM list of genes for the FRDA adult cohort.
(4.67 MB XLS)

Dataset S5 Biologically informative gene sets for the FRDA adult cohort using Gene Set Analysis.
(0.19 MB XLS)

Dataset S6 The association of the DNA damage to predefined gene sets.
(0.18 MB XLS)

Dataset S7 Biologically informative gene sets for FRDA lymphoblastoid data using Gene Set Analysis and compared to FRDA children gene sets.
(0.20 MB XLS)
