# Supplementary figures and images for: Altered Gene Expression and DNA Damage in Peripheral Blood Cells from Friedreich's Ataxia Patients: Cellular Model of Pathology
Source: PLoS Genet. 2010 Jan 15;6(1):e1000812. doi: 10.1371/journal.pgen.1000812 (PMC2799513; doi:10.1371/journal.pgen.1000812)

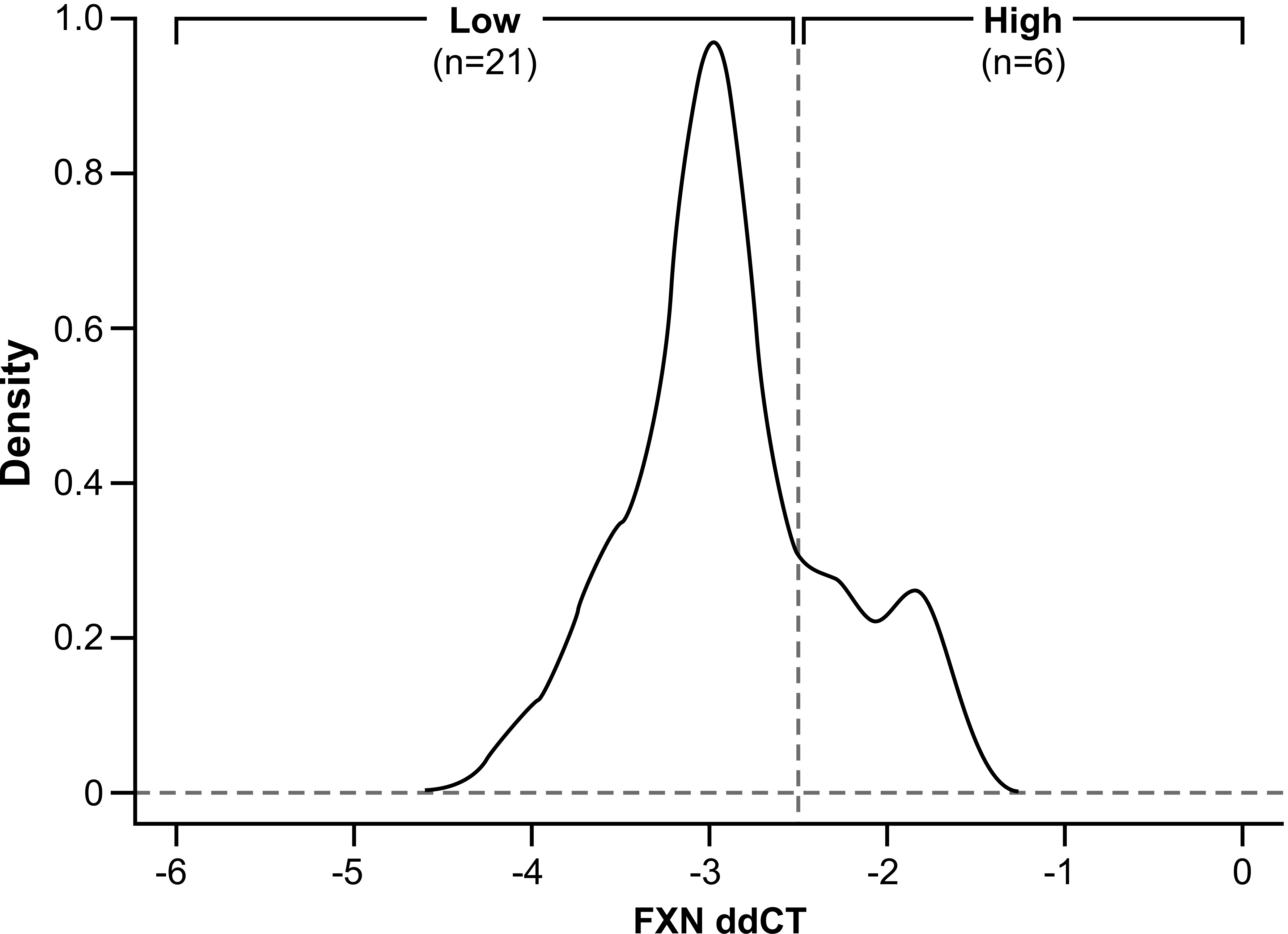

Supplement: Figure S1 — Stratification of Friedreich's ataxia patients based on the distribution of frataxin expression levels. A density plot of frataxin (FXN) expression by Real-time PCR illustrates the distribution of FXN expression over the cases. The x-axis indicates the change in cycle threshold (ddCT) in the cases relative to a pool of the controls. A threshold of −2.5 was selected to split the cases into those with high expression of FXN (6 cases) versus those with relatively low expression of FXN (21 cases). (0.17 MB TIF) [file pgen.1000812.s001.tif]

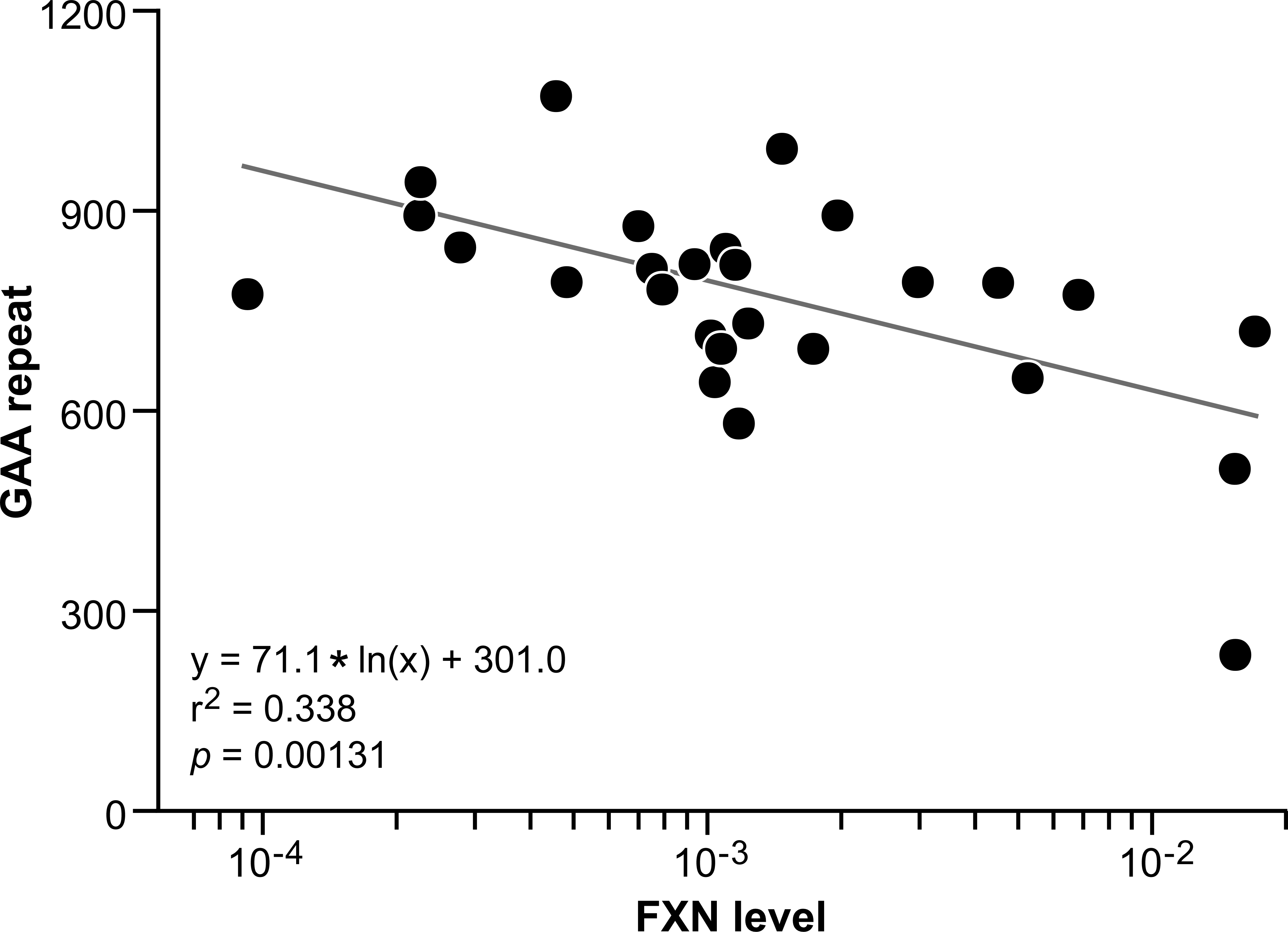

Supplement: Figure S2 — GAA repeat length correlates with frataxin levels. Individual FXN levels were determined for each patient by real-time PCR. A univariate linear model was constructed to test the association of frataxin mRNA levels with short GAA repeats. The short GAA repeat length correlated with mRNA levels (r 2 = 0.338, p = 0.00131). (0.17 MB TIF) [file pgen.1000812.s002.tif]

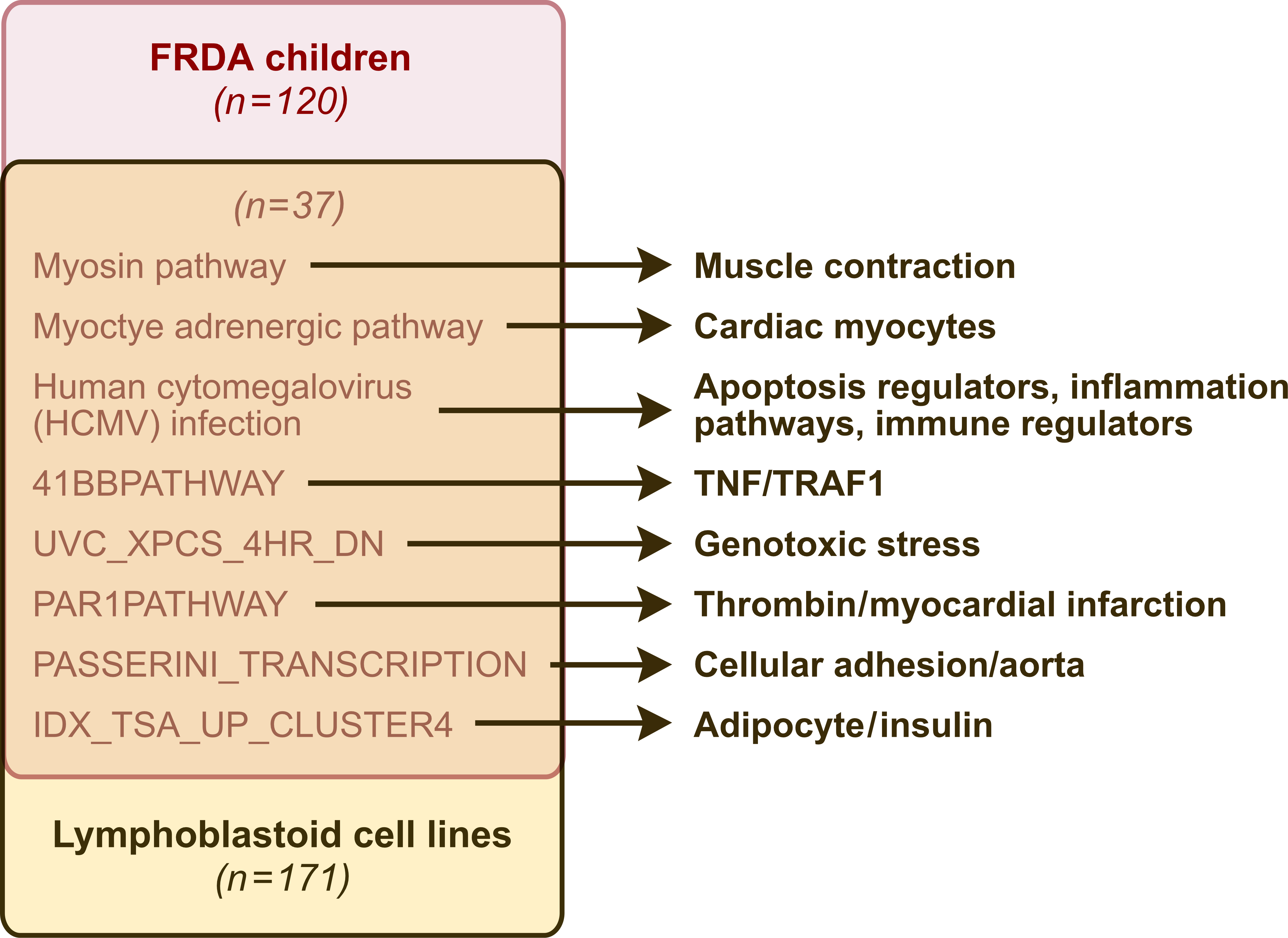

Supplement: Figure S3 — Gene Set Analysis finds gene sets in common between the lymphoblastoid cell line and the FRDA children datasets. Significantly associated gene sets from Gene Set Enrichment Analysis subcatalog C2, a database of 1,684 microarray experiment gene sets, pathways, and other groups of genes, were identified for both the lymphoblastoid cell line and FRDA children datasets. The analysis yielded many biologically informative sets (n = 171, p≤0.05 and n = 120, p≤0.05 for the lymphoblastoids and FRDA children, respectively) with 37 gene sets in common for the two datasets. The Venn diagram displays 8 selected gene sets that associate with both datasets. Descriptions in black loosely summarize the gene set's association to phenotype, cells, tissue, or pathway as described by the authors of origin in the database. (0.99 MB TIF) [file pgen.1000812.s003.tif]

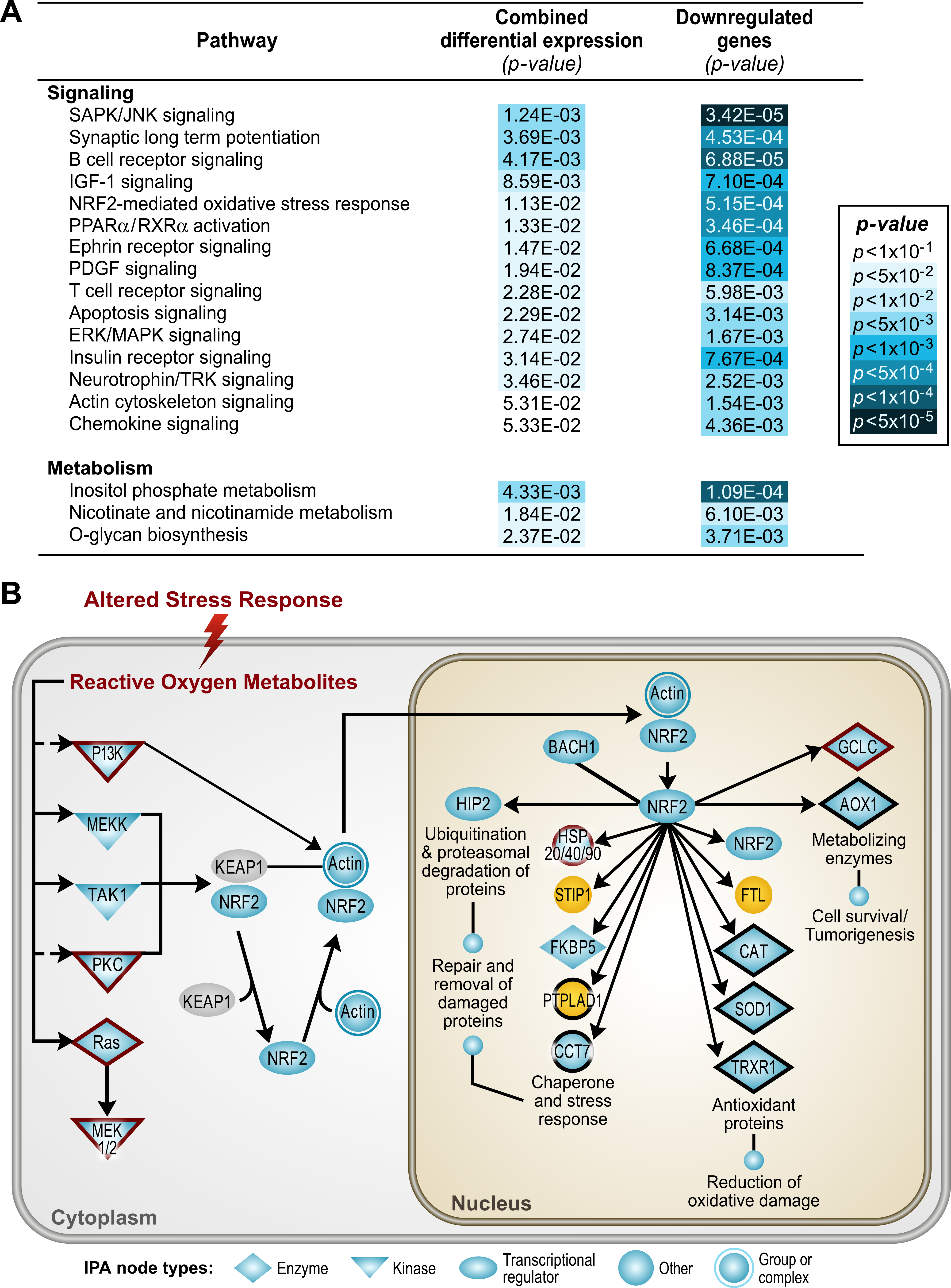

Supplement: Figure S4 — Highly significant pathways responding to frataxin reduction in FRDA patients are driven by downregulation. Significant p-values were calculated by the right-tailed Fisher's Exact test using the entire dataset in the Ingenuity Pathway Analysis program. (A) Significance of the top signaling and metabolic pathways (p-value≥0.05) in the complete list of differentially expressed genes compared to that of downregulated genes only (FDR<0.023%). Upregulated genes only did not reach significance and were not included. (B) Frataxin deficiency downregulates genes involved in the NRF2-mediated oxidative stress pathway (adapted from the Ingenuity Pathway Analysis Knowledge Base). Induced and repressed genes are depicted in yellow and blue, respectively. Genes with no borders are significant in FRDA children, genes with black borders are significant in FRDA adults, and genes with red borders are significant in both. Significant genes in FRDA children not shown: FKBP5 (↓). Significant genes in FRDA adults not shown: ASK1 (↓), MEK5 (↓), JNK1/2 (↓), EIF2AK3 (↓), GSTO1 (↓), GSTA1 (↑), GSTM3 (↑), JUN (↑), MAFG (↓), NQO1 (↑). (3.92 MB TIF) [file pgen.1000812.s004.tif]
