# Supplementary material for: Altered Gene Expression and DNA Damage in Peripheral Blood Cells from Friedreich's Ataxia Patients: Cellular Model of Pathology
Source: PLoS Genet. 2010 Jan 15;6(1):e1000812. doi: 10.1371/journal.pgen.1000812 (PMC2799513; doi:10.1371/journal.pgen.1000812)
Supplement: Table S1 — Demographics for Friedreich's ataxia children involved in gene expression analysis of peripheral blood. (0.03 MB DOC) [file pgen.1000812.s005.doc]

| **Total Subjects (n=28)** | |
| --- | --- |
| Age, mean (SD), y | 13.5 (2.3) |
| Males, No. (%) | 16 (57) |
| GAA length-allele 1, mean (SD) | 774 (158) |
| GAA length-allele 2, mean (SD) | 1022 (201) |
| Age of diagnosis, mean (SD), y | 10.1 (2.4) |
| Age of onset, mean (SD), y | 7.5 (3.1) |
| Disease Duration, mean (SD), y | 6.0 (3.5) |
| ADL score, mean (SD) | 14.1 (4.8) |
| ICARS score, mean (SD) | 41.7 (13.6) |
| FARS score, mean (SD) | 50.9 (16.9) |
| **Total Controls (n=10)** | |
| Age, mean (SD), y | 20.3 (1.4) |
| Males, No. (%) | 8 (80) |

**Table S1.**  Demographics for Friedreich’s ataxia children involved in gene expression analysis of peripheral blood.
