# Supplementary material for: Altered Gene Expression and DNA Damage in Peripheral Blood Cells from Friedreich's Ataxia Patients: Cellular Model of Pathology
Source: PLoS Genet. 2010 Jan 15;6(1):e1000812. doi: 10.1371/journal.pgen.1000812 (PMC2799513; doi:10.1371/journal.pgen.1000812)
Supplement: Table S2 — Demographics for Friedreich's ataxia adult subjects involved in gene expression analysis of peripheral blood. (0.03 MB DOC) [file pgen.1000812.s006.doc]

| **Total Subjects (n=14)** | |
| --- | --- |
| Age, mean (SD), y | 36.8 (15.9) |
| Males, No. (%) | 7 (50) |
| GAA length-allele 1, mean (SD) | N/A |
| GAA length-allele 2, mean (SD) | N/A |
| Age of diagnosis, mean (SD), y | N/A |
| Age of onset, mean (SD), y | N/A |
| Disease Duration, mean (SD), y | N/A |
| ADL score, mean (SD) | N/A |
| ICARS score, mean (SD) | N/A |
| FARS score, mean (SD) | N/A |
| **Controls (n=15)** | |
| Age, mean (SD), y | 25.4 (9.5) |
| Males, No. (%) | 10 (67) |

**Table S2.** Demographics for Friedreich’s ataxia adult subjects involved in gene expression analysis of peripheral blood.
