# Supplementary material for: Altered Gene Expression and DNA Damage in Peripheral Blood Cells from Friedreich's Ataxia Patients: Cellular Model of Pathology
Source: PLoS Genet. 2010 Jan 15;6(1):e1000812. doi: 10.1371/journal.pgen.1000812 (PMC2799513; doi:10.1371/journal.pgen.1000812)
Supplement: Table S3 — The complete list of GO terms associated to the 228 significant genes in common between the FRDA children and FRDA adults. (0.06 MB DOC) [file pgen.1000812.s007.doc]

| **Biological Process Term** | **Gene#** | **%** | ***p*-Value** |
| --- | --- | --- | --- |
| Biopolymer modification | 36 | 16.98% | 6.98E-05 |
| Protein modification | 34 | 16.04% | 2.11E-04 |
| Ubiquitin cycle | 16 | 7.55% | 2.82E-04 |
| Biopolymer metabolism | 49 | 23.11% | 2.93E-04 |
| Cellular physiological process | 130 | 61.32% | 0.001292853 |
| Primary metabolism | 95 | 44.81% | 0.010047576 |
| Protein ubiquitination | 8 | 3.77% | 0.013332587 |
| Cellular metabolism | 97 | 45.75% | 0.013816613 |
| Cell cycle | 15 | 7.08% | 0.016291689 |
| Modification-dependent protein catabolism | 6 | 2.83% | 0.018942198 |
| Ubiquitin-dependent protein catabolism | 6 | 2.83% | 0.018942198 |
| RNA splicing | 6 | 2.83% | 0.022615388 |
| Nucleobase, nucleoside, nucleotide and nucleic acid metabolism | 49 | 23.11% | 0.024651005 |
| Cellular macromolecule metabolism | 46 | 21.70% | 0.025380996 |
| Metabolism | 101 | 47.64% | 0.029117486 |
| Cellular protein metabolism | 45 | 21.23% | 0.030274442 |
| Macromolecule metabolism | 61 | 28.77% | 0.034670416 |
| Regulation of biological process | 50 | 23.58% | 0.039158158 |
| Cellular process | 143 | 67.45% | 0.04095028 |
| Proteolysis during cellular protein catabolism | 6 | 2.83% | 0.042232244 |
| Cellular protein catabolism | 6 | 2.83% | 0.043122072 |
| RNA splicing, via transesterification reactions with bulged adenosine as nucleophile | 5 | 2.36% | 0.043414678 |
| RNA splicing, via transesterification reactions | 5 | 2.36% | 0.043414678 |
| Nuclear mRNA splicing, via spliceosome | 5 | 2.36% | 0.043414678 |
| Regulation of progression through cell cycle | 10 | 4.72% | 0.053480914 |
| Regulation of cell cycle | 10 | 4.72% | 0.054060984 |

**Table S3.** The complete list of GO terms associated to the 228 significant genes in common between the FRDA children and FRDA adults.
