# Supplementary material for: Altered Gene Expression and DNA Damage in Peripheral Blood Cells from Friedreich's Ataxia Patients: Cellular Model of Pathology
Source: PLoS Genet. 2010 Jan 15;6(1):e1000812. doi: 10.1371/journal.pgen.1000812 (PMC2799513; doi:10.1371/journal.pgen.1000812)
Supplement: Table S4 — Ingenuity Pathway Analysis. Ingenuity Knowledge Base biological function categories and pathways associated to the overlap of 228 significant genes from the FRDA children and FRDA adults, respectively (p≥0.05). (0.11 MB RTF) [file pgen.1000812.s008.rtf]

      Table S4.  Ingenuity Pathway Analysis of significant genes that overlap between children and adults with FRDA. 
Diseases and Disorders	Genes	
Cancer	SIAH1, TNFAIP8, SFRS2, TXNIP, RECK, MAPK8, E2F3, RNF6, TP53BP2, C19ORF2, HNRPK, SGMS1, ATF2, RP6-213H19.1, PAQR3, F2RL1, ABTB1, MYB, MAP2K1, CDK2, ITGA4, PRNP	
Cardiovascular Disease	BNIP2, SFRS2, TXNIP,  MYB, ITGA4	
Viral Function	CHUK, PRNP	
Hematological Disease	TXNIP, MAPK8, MYB, MAP2K1, CDK2, ITGA4, ATF2	
Immunological Disease	MAPK8, MYB, CDK2, ITGA4	
Molecular and Cellular Function	Genes	
Cell Cycle	SIAH1, SFRS2, CCNC, BTG3, TXNIP, MAPK8, E2F3, TP53BP2, ATF2, SESN1, ABTB1, PCTK2, USP16, MYB, CHUK, MAP2K1, RAD21, CDK2	
Cell Death	TNFAIP8, SIAH1, GCLC, E2F3, SGMS1, ATP7A, F2RL1, MYB, CHUK, MAP2K1, RAD21, PRNP, ITGA4, TXNIP, MAPK8, CCAR1, TP53BP2, SLC25A24, ATF2, RP6-213H19.1, ROCK1, BNIP2, PPM1B, CDK2, ABCE1	
Cellular Growth and Proliferation	TNFAIP8, SIAH1, SFRS2, BTG3, TXNIP, RECK, MAPK8, E2F3, SGMS1, HNRPK, ATF2, RP6-213H19.1, F2RL1, ABTB1, ACP1, MYB, MAP2K1, CDK2, SCTR, ITGA4	
Cell Morphology	ROCK1, F2RL1, ACP1, MAPK8, MYB, CDC42SE2, GOLGA5, E2F3, MAP2K1, RNF6, ITGA4	
Cellular Function and Maintenance	ROCK1, ATP7A, PEX3, RAB5A, PRNP	
Physiological System Development & Function	Genes	
Reproductive System Development and Function	SIAH1, ABTB1, SCTR, CDK2, ATF2	
Cardiovascular System Development and Function	ROCK1, BNIP2, SFRS2, ATP7A, F2RL1, TXNIP, ACP1, MAPK8, MYB, ITGA4	
Visual System Development and Function	ROCK1, MAPK8	
Connective Tissue Development and Function	TNFAIP8, SFRS2, BTG3, TXNIP, MAPK8, E2F3, ATF2, ROCK1, F2RL1, ACP1, CDC42SE2, CHUK, MAP2K1, CDK2, ITGA4	
Skeletal and Muscular System Development and Function	BNIP2, SFRS2, F2RL1, TXNIP, RECK, MYB, MAPK8, CHUK, MAP2K1	
Signaling Pathways	Genes	
Apoptosis Signaling	ROCK1, MAPK8, CHUK, CAPN7, MAP2K1, CDK2	
SAPK/JNK Signaling	MAPK8, MAP4K5, MAP2K1, CDK2, HNRPK, ATF2	
Integrin Signaling	ROCK1, MAPK8, PPP1R12A, CAPN7, MAP2K1, ITGA4	
Ephrin Receptor Signaling	ROCK1, ACP1, MAPK8, MAP2K1, CDK2, ATF2	
ERK/MAPK Signaling	PPP2CB, MAPK8, PPP1R12A, MAP2K1, CDK2, TF2	
PI3K/AKT Signaling	PPP2CB, MAPK8, CHUK, AP2K1, CDK2	
Neurotrophin/TRK Signaling	MAPK8, MAP2K1, ATF2	
PDGF Signaling	ACP1, MAPK8, MAP2K1	
Chemokine Signaling	MAPK8, PPP1R12A, MAP2K1	
FGF Signaling	MAPK8, MAP2K1, ATF2	
Protein Ubiquitination Pathway	USP16, UBR1, UCHL5, USP1	
B Cell Receptor Signaling	MAPK8, CHUK, MAP2K1, ATF2	
Metabolic Pathways	Genes	
Oxidative Phosphorylation	ATP7A, NDUFA5, COX11, ATP5B	
Purine Metabolism	PRPS2, ATP5B, DCK, GDA,  POLR2B, DHX15	
Inositol Phosphate Metabolism	MAPK8, PLCL2, MAP2K1, CDK2	
Nicotinate and Nicotinamide Metabolism	MAPK8, MAP2K1, CDK2	
