# Supplementary material for: Altered Gene Expression and DNA Damage in Peripheral Blood Cells from Friedreich's Ataxia Patients: Cellular Model of Pathology
Source: PLoS Genet. 2010 Jan 15;6(1):e1000812. doi: 10.1371/journal.pgen.1000812 (PMC2799513; doi:10.1371/journal.pgen.1000812)
Supplement: Table S5 — Gene Set Analysis of common genes. Enriched gene sets associated to significant genes (SAM) that overlap between FRDA children and FRDA adults. (0.04 MB DOC) [file pgen.1000812.s009.doc]

**Table S5.** Gene Set Analysis of common genes between the children and adult FRDA cohorts.
