# Supplementary material for: Altered Gene Expression and DNA Damage in Peripheral Blood Cells from Friedreich's Ataxia Patients: Cellular Model of Pathology
Source: PLoS Genet. 2010 Jan 15;6(1):e1000812. doi: 10.1371/journal.pgen.1000812 (PMC2799513; doi:10.1371/journal.pgen.1000812)
Supplement: Table S6 — Demographics for Friedreich's ataxia children involved in DNA damage analysis of peripheral blood. (0.03 MB DOC) [file pgen.1000812.s010.doc]

| **Total Subjects (n=47)** | |
| --- | --- |
| Age, mean (SD), y | 13.4 (2.4) |
| Males, No. (%) | 24 (51) |
| GAA length-allele 1, mean (SD) | 788 (131) |
| GAA length-allele 2, mean (SD) | 963 (270) |
| Age of diagnosis, mean (SD), y | 9.7 (2.5) |
| Age of onset, mean (SD), y | 7.6 (2.9) |
| Disease Duration, mean (SD), y | 5.7 (3.7) |
| ADL score, mean (SD) | 13.7 (4.6) |
| ICARS score, mean (SD) | 40.3 (14.3) |
| FARS score, mean (SD) | 51.5 (16.8) |
| **Controls (n=15)** | |
| Age, mean (SD), y | 31.8 (8.5) |
| Males, No. (%) | 14 (93) |

**Table S6.** Demographics for Friedreich’s ataxia children involved in DNA damage analysis of peripheral blood.
