# Supplementary material for: Altered Gene Expression and DNA Damage in Peripheral Blood Cells from Friedreich's Ataxia Patients: Cellular Model of Pathology
Source: PLoS Genet. 2010 Jan 15;6(1):e1000812. doi: 10.1371/journal.pgen.1000812 (PMC2799513; doi:10.1371/journal.pgen.1000812)
Supplement: Table S7 — Gene names and corresponding IDs. (0.19 MB RTF) [file pgen.1000812.s011.rtf]

Table S7. All genes and their corresponding gene ENTREZ identification numbers.

Gene Name	Entrez Gene ID	
ACTN4	81	
ADAM23	8745	
ADSS	159	
AP2B1	163	
APRT	353	
ATM	472	
ATP5G2	517	
C10orf88	80007	
CCAR1	55749	
CCND3	896	
CD69	969	
CDC42BPB	9578	
CEACAM6	4680	
CES1	1066	
CFL1	1072	
COX23	856516	
CPOX	1371	
CRIP2	1397	
CTSF	8722	
CXCL11	6373	
DHFRL1	200895	
DSG1	1828	
ERCC1	2067	
FANCJ	83990	
FAU	2197	
FHL2	2274	
FPR1	2357	
FXN	2395	
GADD45A	1647	
GLI2	2736	
GSPT2	23708	
HAT1	8520	
HIPK2	28996	
HLA-C	3107	
HSPC152	51504	
IFITM1	8519	
IMPACT	55364	
IRX2	153572	
ITGA4	3676	
ITGAE	3682	
KIAA0907	22889	
LILRB3	11025	
LOC51334	51334	
MAN1B1	11253	
MAP4	4134	
MRPS14	63931	
MRPS14	63931	
MRPS24	64951	
MRPS28	28957	
MS4A2	2206	
MTHFR	4524	
MUTYH	4595	
NDUFA5	4698	
NGFRAP1	27018	
NHP2L1	4809	
NME3	4832	
NRF2	4780	
NXT1	29107	
ONECUT1	3175	
PAPSS2	9060	
PEX6	5190	
PMAIP1	5366	
POU2F2	5452	
PRI2	853821	
RAD3	856918	
RAD52	854976	
RPL13A	23521	
RPL18A	6142	
RPL19	6143	
RPL22	6146	
RPL23A	6147	
RPL24	6152	
RPL26L1	51121	
RPL27	6155	
RPL28	6158	
RPL29	6159	
RPL36	25873	
RPL37	6167	
RPL37A	6168	
RPL3L	6123	
RPLP2	6181	
RPS10	6204	
RPS12	6206	
RPS13	6207	
RPS14	6208	
RPS15	6209	
RPS19	6223	
RPS20	6224	
RPS23	6228	
RPS26	6231	
RPS27A	6233	
RPS28	6234	
RPS3	6188	
RPS4X	6191	
RPS5	6193	
S100A12	6283	
S100A9	6280	
S100B	6285	
SERPINC1	462	
SESN1	27244	
SGCE	8910	
SLC27A5	10998	
SLC5A4	6527	
STX6	10228	
TBX3	6926	
TLR4	7099	
TMEM9	252839	
TP53	7157	
UXT	8409	
XPD	2068	
XPF	2072	
XPO7	23039	
