# Supplementary material for: Altered Gene Expression and DNA Damage in Peripheral Blood Cells from Friedreich's Ataxia Patients: Cellular Model of Pathology
Source: PLoS Genet. 2010 Jan 15;6(1):e1000812. doi: 10.1371/journal.pgen.1000812 (PMC2799513; doi:10.1371/journal.pgen.1000812)
Supplement: Table S8 — Demographic data for FRDA and control lymphoblastoid cell lines. Data from a previous lymphoblastoid gene expression analysis [45] was of limited use due to replicate noise, difference in microarray platform (they used Affymetrix), and only one affected and one control lymphoblastoid comparison; thus, few comparisons could be drawn between our lymphoblastoid data or our cohort data with their data. (0.07 MB RTF) [file pgen.1000812.s012.rtf]

Cell Line	Repository #	Race	# of GAA Repeats	
Control 14 yr-old	GM15851	Caucasian	Normal	
Control 21 yr-old	GM14650	Caucasian	Normal	
Control 29 yr-old	GM09631	Caucasian	Normal	
Control 51 yr-old	GM01990	Caucasian	Normal	
Control 37 yr-old	GM10450	Caucasian	Normal	
Control 23 yr-old	GM11494	Caucasian	Normal	
Control 29 yr-old	GM07496	Caucasian	Normal	
FRDA 13 yr-old	GM15850	Caucasian	650-1030	
FRDA 22 yr-old	GM16201	Caucasian	450-640	
FRDA 30 yr-old	GM04079	Caucasian	340-420	
FRDA 29 yr-old	GM16203	Caucasian	670-830	
FRDA 53 yr-old	GM16204	Caucasian	380-830	
FRDA 39 yr-old	GM16210	Caucasian	580-580	
FRDA 14 yr-old	GM16197	N/A	760-830	
FRDA 23 yr-old	GM16243	Caucasian	670-1170	
FRDA 30 yr-old	GM16205	Caucasian	530-530	
Table S8.  Demographic data for FRDA and control lymphoblastoid cell lines. 
